# Upregulation of Prostaglandin Receptor EP_1_ Expression Involves Its Association with Cyclooxygenase-2

**DOI:** 10.1371/journal.pone.0091018

**Published:** 2014-03-10

**Authors:** Rapita Sood, Galit Flint-Ashtamker, Dafna Borenstein, Liza Barki-Harrington

**Affiliations:** Department of Human Biology, Faculty of Natural Sciences, University of Haifa, Mt. Carmel, Haifa, Israel; University of Oslo, Norway

## Abstract

While many signals cause upregulation of the pro-inflammatory enzyme cyclooxygenase -2 (COX-2), much less is known about mechanisms that actively downregulate its expression. We have recently shown that the prostaglandin EP_1_ receptor reduces the expression of COX-2 in a pathway that facilitates its ubiquitination and degradation via the 26S proteasome. Here we show that an elevation of COX-2 intracellular levels causes an increase in the endogenous expression of prostaglandin EP_1_. The increase in EP_1_ levels does not occur at the transcriptional level, but is rather associated with complex formation between the receptor and COX-2, which occurs both in *vitro* and in mammalian tissues. The EP_1_-COX-2 complex is disrupted following binding of arachidonic acid to COX-2 and accompanied by a parallel reduction in EP_1_ levels. We propose that a transient interaction between COX-2 and EP_1_ constitutes a feedback loop whereby an increase in COX-2 expression elevates EP_1_, which ultimately acts to downregulate COX-2 by expediting its proteasomal degradation. Such a post translational mechanism may serve to control both the ligand-generating system of COX-2 and its reception system.

## Introduction

Lipid metabolites of arachidonic acid (AA) play central roles in the regulation of key physiological functions such as immunity, inflammation, gastrointestinal integrity and cardiovascular homeostasis [1]. AA is cleaved from membrane phospholipids by phospholipase A_2_, immediately followed by a two-step catalysis into H_2_ prostaglandin endoperoxide (PGH_2_) by the rate-limiting enzyme cyclooxygenase (COX). PGH_2_ gives rise to five biologically active prostanoids (PGD_2_, PGE_2_, PGF_2_, PGI_2_ and TXA_2_) by specific prostaglandin synthases residing in different tissues [2], [3]. Once formed, these bioactive lipids exert their cellular functions by activating receptors from the super-family of rhodopsin-like G-protein coupled receptors (GPCRs).

Among prostanoids, PGE_2_ is the major product of AA metabolism, most common across species, and the most versatile in its functions. It is known to play several important physiological roles (e.g. facilitation of ovulation and implantation, regulation of smooth muscle contractility), as well as pathophysiological ones (*e.g*. mediation of inflammation, tumor growth and invasion) [1]. Its actions are mediated through activation of four subtypes of prostaglandin E_2_ (EP) GPCRs, designated EP_1–4_, each encoded by a different gene and respond differently to selective agonists and antagonists [1], [4]. Although all four receptors bind PGE_2_ with a higher affinity than other prostanoids, they differ substantially in their intracellular signaling, desensitization and internalization patterns [5], [6], and while the signaling pathways of EP_2–4_ are well studied, those of EP_1_ are partially characterized [7], [8].

As the primary source of PGE_2_, the levels and enzymatic activity of COXs are critical for EP receptor signaling. COXs exist in two main isoforms, COX-1 and COX-2, and although encoded by two separate genes, they share a high degree of sequence homology and display similar catalytic mechanisms [2], [3]. However, both isoforms markedly differ in expression and biological functions. COX-1 is expressed almost ubiquitously, fulfills many housekeeping functions (*e.g*. female reproduction, gastric protection and cardiovascular homeostasis) [9], [10], [11], and is a relatively stable protein. Conversely, COX-2 expression undergoes rapid and transient increase by a broad range of pathological stimuli [11], [12] and its expression is regulated by at least six different promoter-response elements [13], [14].

While many signals cause COX-2 upregulation, much less is known about mechanisms that actively downregulate its expression. In this regard, we have recently found that the EP_1_ receptor reduces the expression of COX-2, through a mechanism that does not involve classical receptor signaling. Instead, we showed that EP_1_ forms a complex with COX-2 and facilitates its ubiquitination, thereby accelerating its degradation through the proteasomal pathway [15]. Since these findings proposed a new role for the EP_1_ receptor in resolving inflammation by downregulation of COX-2 levels, we tested the hypothesis that elevated expression of COX-2 may upregulate the expression of EP_1_ receptors, thus constituting a feedback loop that ultimately serves to downreglate COX-2. For this we tested the effect of COX-2 overexpression on the levels of endogenous EP_1_ and how binding of AA to COX-2 affects the interaction between them. Finally, we documented interactions between COX-2 and EP_1_ in various mammalian organs, supporting a possible physiological relevance for this interaction.

## Materials and Methods

### Materials

Goat polyclonal anti-COX-2 (human), rabbit polyclonal anti-pERK and mouse monoclonal anti-ERK were obtained from Santa Cruz Biotechnologies (Santa Cruz, CA). Rabbit polyclonal anti-EP_1_, EP_2_, EP_3_ and EP_4_ receptors (human) were from Cayman Chemical (Ann Arbor, MI) as was arachidonic acid. 17-phenyl-trinor prostaglandin E_2_ (17E_2_) was from Biomol -Enzo Life Sciences (Pharmingdale, NY). Horseradish peroxidase-conjugated bovine anti-goat IgG, goat anti-rabbit IgG, and goat anti-mouse IgG were obtained from Jackson ImmunoResearch Laboratories (West Grove, PA). All other reagents were standard laboratory grade.

### Cell culture and transfection

HEK-293 cells were obtained from the ATCC and used between passages 10–30. Bovine aortic endothelia cells (BAEC) were obtained from Prof. Israel Vlodavski, The Hebrew University, Jerusalem, Israel [16]. Transient transfections were carried out at sub confluent (70–80%) monolayers using PolyJet (SignaGen Laboratories) at a ratio of 1∶3 cDNA: PolyJet, according to the manufacturer's instructions. All samples contained the same amount of total cDNA.

### cDNA Constructs

EP_1_ cDNA was gift of Prof. Barry Ashby, Temple University School of Medicine. pcDNA5/FRT/TO encoding human COX-2 and G533ACOX-2 were gift from Prof. William L. Smith, University of Michigan.

### Immunoprecipitation and immunoblotting

Monolayers in 100-mm culture dishes were washed twice with ice-cold PBS and lysed in 1 mL RIPA/SDS buffer (50 mM Tris pH 8, 150 mM NaCl, 5 mM EDTA, 1% v/v NP-40, 0.5%, 0.5% w/v deoxycholic acid, 0.1% w/v SDS, 10 mM NaF, 0.1 mM PMSF and Complete Protease Inhibitor cocktail tablets (Roche)), exactly as described before (15).

### RNA isolation and Quantitative PCR

Total RNA was isolated as we have described before [15]. Quantitative real-time PCR was performed with a StepOnePlus™Real-Time PCR System using Fast SYBR Green technology (Applied Biosystems, USA) using the forward primer 5′- CGCTGCAGGGAGGTAGAG-3′ and the reverse primer 5′-ATGGTGGTGTCGTGCATCT -3′ for EP_1_, and the forward primer 5′-CCTTGCCTTTCACGATTTTTG-3′ and reverse primer 5′-TAAGAGCTTGGAGGTCCCATTTT-3′ for EP_2_. Results were analyzed using StepOne software (Applied Biosystems). COX-2 mRNA abundance was normalized to the hypoxanthine-guanine phosphoribosyltransferase (HPRT) gene as an endogenous control.

### Animals and Tissue Homogenization

Sprague Dawly rats weighing 200–300 g were used in the study. Animals were housed under diurnal lighting conditions and allowed food and tap water *ad libitum*. On day of experiment, rats were sacrificed following anesthesia (Isoflurane, Abbott Laboratories, Abbott Park, IL) and organs were harvested and immediately frozen at −80°C pending analysis. Tissues were placed in a glass Teflon tissue homogenizer and homogenized by 16–20 strokes in 1 mL RIPA/SDS and protease inhibitors, and immunoprecipitation was carried out as above. All experimental protocols were approved by the Animal Care and Use Committee of the University of Haifa.

### Statistical analysis

Unless otherwise stated, statistical significance was determined by one-way ANOVA. Post-hoc analysis was performed with Tukey multi-comparison test when appropriate. p values <0.05 were considered significant.

## Results

**Figure 1:**
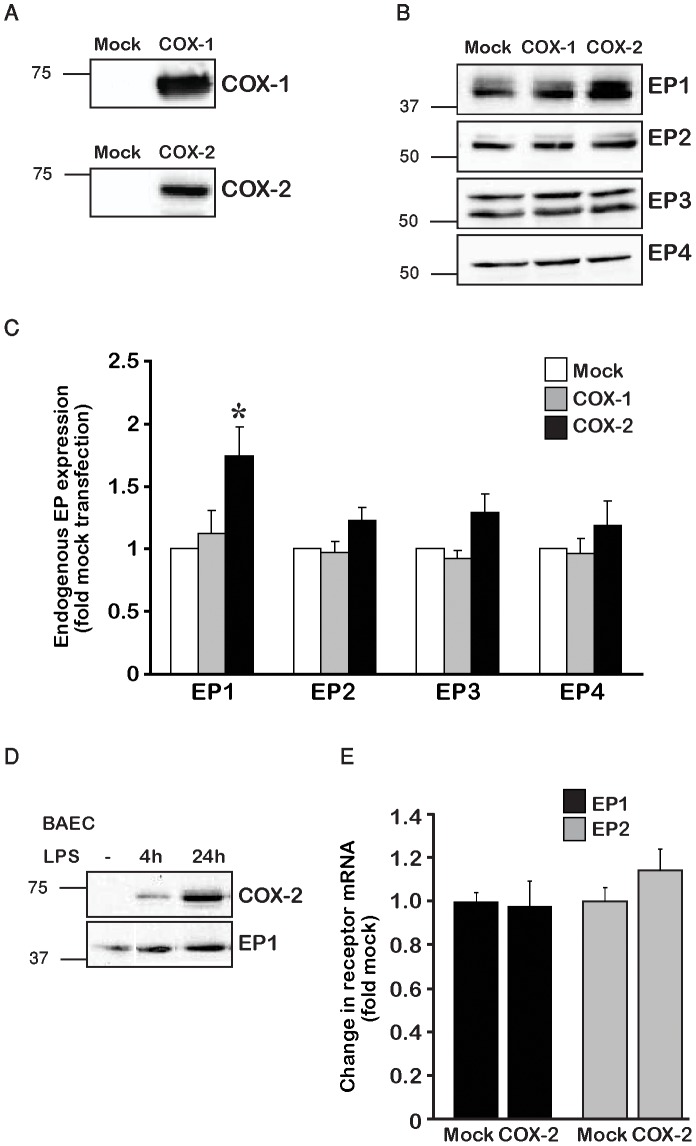
Overexpression of COX-2 raises endogenous levels of EP_1_. *A*, HEK 293 cells do not express detectable levels of COXs. Representative immunoblot of cell lysates transfected with 0.5 µg empty vector (pcDNA3.1, Mock) or wt COX-1 (upper panel) or COX-2 (lower panel). *B*, Effect of COX-1 and COX-2 overexpression on EP receptor levels. Representative immunoblots of cells were transfected with 0.5 µg mock, COX-1 or COX-2. Endogenous expression of EP receptor levels was determined using specific antibodies. *C*, Summary graph of the effect of COX overexpression on EP levels n = 7–9 different experiments. Shown are mean + SEM *p<0.05 vs. Mock transfection. *D*, Bovine aortic endothelial cells (BAEC) were treated with 1 µM LPS for the indicated times. Lysates were collected and probed for COX-2 and EP_1_ levels. *E*, HEK 293 cells were transfected with 0.5 µg COX-2 or mock and samples were analyzed for content of endogenously expressing EP_1_ or EP_2_ mRNA using real-time PCR. n = 3 independent experiments performed in triplicates. P = 0.386 vs. mock.

Our previous research had shown that elevated levels of EP_1_ downregulate the expression of COX-2 in a mechanism that does not involve receptor activation [15]. In the current study we sought to test whether elevated levels of COX-2, such that occur in many pathological conditions, may reciprocally affect the expression of EP receptors. For this, we used HEK 293 cells that lack detectable levels of endogenous COXs in the absence of transfection (Fig. 1A), but show detectable levels of all four types of endogenously expressed EP receptors (Fig. 1B). We transfected cells with either COX-1 or COX-2 and measured the effect of this overexpression on the levels of EP receptors. Analysis revealed that overexpression of COX-1 did not affect the expression of any of the EP receptors (Fig. 1B). In contrast, while overexpression of COX-2 had no effect on the levels of EP_2_, EP_3_ or EP_4_, it caused a marked increase in the expression of endogenous EP_1_ (∼1.8 fold) (Fig. 1B and 1C). To corroborate these results in another system, we stimulated bovine aortic endothelial cells (BAEC) with the pro-inflammatory agent LPS for 4 and 24 h and measured COX-2 and EP_1_ levels. As shown in Fig. 1D, in the absence of LPS, BAEC do not express any detectable levels of COX-2 and relatively low levels of EP_1_. However, exposure of the cells to LPS caused a gradual increase in COX-2 expression, which was mirrored by elevation in EP_1_ expression.

To determine whether the increase in EP_1_ protein expression was a result of elevated transcription, we performed real-time PCR measurements of EP_1_ mRNA in HEK 293 cells transfected with either mock or COX-2 cDNA. As shown in Fig. 1E, transfection of COX-2 did not change the mRNA levels of the receptor, suggesting that the elevation of EP_1_ protein expression by COX-2 occurs at a post-transcriptional level.

**Figure 2:**
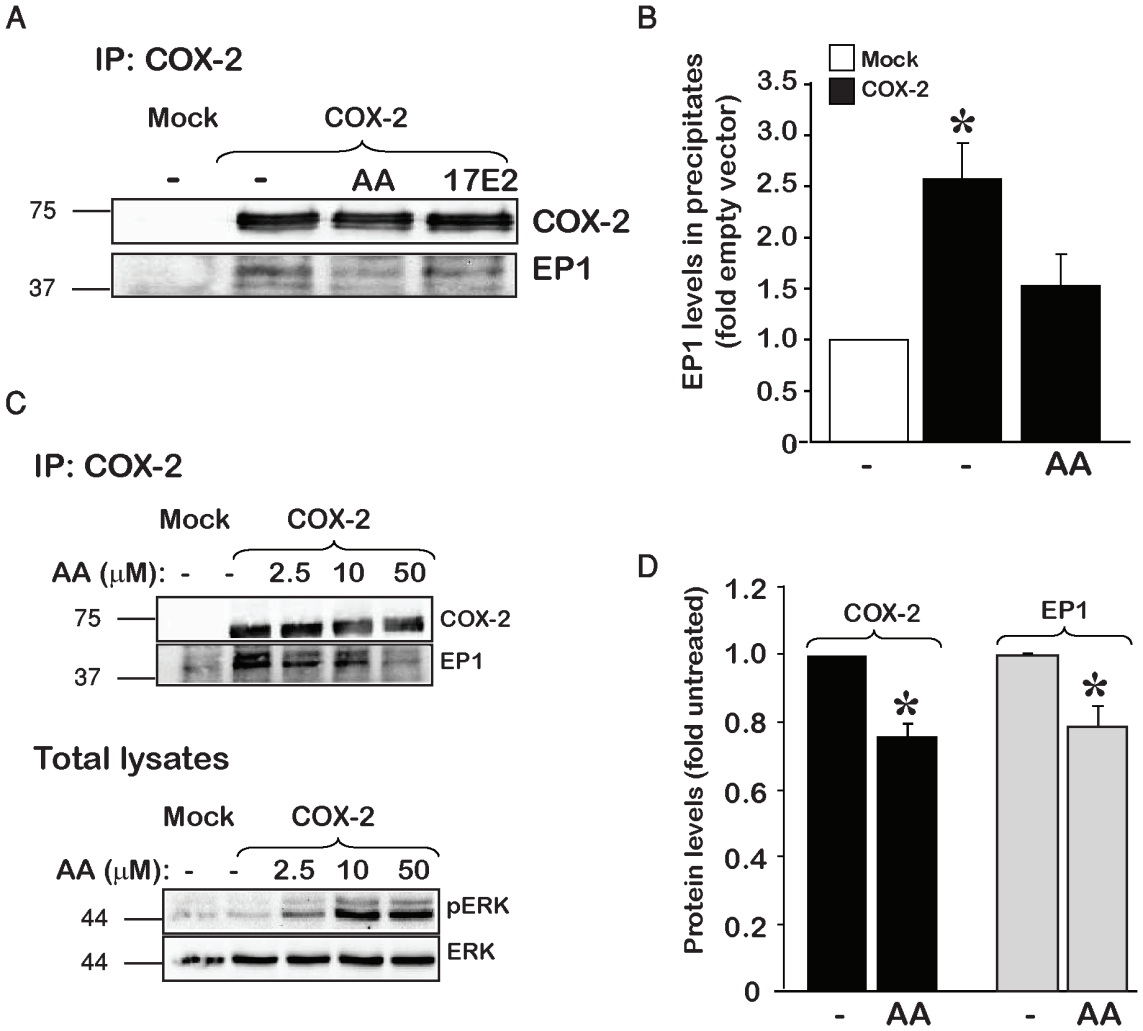
The interaction between COX-2 and EP_1_ is disrupted in the presence of AA. *A*, COX-2 was immunoprecipitated from HEK 293 cells, transfected with Mock or COX-2 and treated with either vehicle (ethanol), AA (50 µM) or the EP_1_ agonist 17E_2_ (1 µM) for 15 min. Blots were probed for COX-2 and EP_1_ using specific antibodies. *B*, Summary graph of n = 5 independent experiments showing a reduction in the levels of EP_1_ co-immunoprecipitated with COX-2 following treatment with AA. *p<0.05 vs. Mock transfection. *C*, Representative immunoblot of n = 3 independent experiments depicting the dose-dependent effect of AA on the interaction of COX-2 with EP_1_. Cells were transfected with either mock or COX-2, and 16 h post-transfection treated with 2.5, 10 or 50 µM AA for 15 min prior to immunoprecipitation. Immunoprecipitate were probed for COX-2 and EP_1_ levels. Total lysates from the same samples were probed first for phosho-ERK followed by total ERK levels using specific antibodies. *D*, prolonged exposure to AA, reduces COX-2 and EP_1_ levels. Cells overexpressing COX-2 were treated with 10 µM AA for 1.5–2 h, samples were collected and probed for levels of COX-2 and EP_1_. n = 5 independent experiments, *p<0.05 vs. untreated.

We have previously shown that COX-2 interacts with endogenous EP_1_ in both normal human dermal fibroblasts and in HEK 293 cells [15]. Here we tested whether the interaction between the two proteins is involved in COX 2-mediated increase in EP_1_ expression, and if it is affected by the conformation of either EP_1_ or COX-2 following binding of ligand or substrate, respectively. For this we measured the interaction of overexpressed COX-2 with endogenous EP_1_ under conditions of non-stimulated enzyme and following a short exposure of COX-2 to AA or to the EP_1_ agonist 17E_2_. A brief exposure to AA was chosen because while it is sufficient to activate the enzyme, it does not cause a reduction in its levels by suicide inactivation [3]. As shown in Fig. 2A, only cells overexpressing COX-2, without stimulus, showed the presence of EP_1_ in the COX-2 precipitates. Activation of the receptor by the selective agonist 17E_2_ did not affect the complex, but treatment with AA significantly decreased the amount of EP_1_ that co-precipitated with COX-2 (Fig. 2A and 2B). To further strengthen the possibility that the decrease in association between the two proteins is due to activation of COX-2 by AA, we performed the same experiment under increasing concentrations of AA and found that the association of COX-2 and EP_1_ was in reverse correlation to the amount of AA, with decreased association as the amounts of AA were increased (Fig. 2C upper panel). Since catalysis of AA by COX-2 yields different metabolites that activate the ERK signaling pathway through numerous prostanoid receptors including EP_1_ [17], we performed concomitant measurements of phosho-ERK in the same samples as an indirect indication of COX-2 activity. As shown in Fig. 2C (lower panel), a gradual increase in AA caused a parallel activation of ERK, suggesting that COX-2 was indeed activated.

Since the presence of AA decreased the interaction between the two proteins, we next sought to test whether prolonged exposure to AA also affects the total levels of endogenously expressed EP_1_. For this we stimulated COX 2-expressing cells with AA for 1.5–2 hours and measured the levels of both COX-2 and EP_1_. Exposure of COX-2 to AA caused a ∼20% reduction in the levels of COX-2. Assessment of EP_1_ levels in the same samples showed a parallel reduction in the expression levels of the receptor (Fig. 2D), further supporting a connection between the expression levels of COX-2 and EP_1_ receptor.

**Figure 3:**
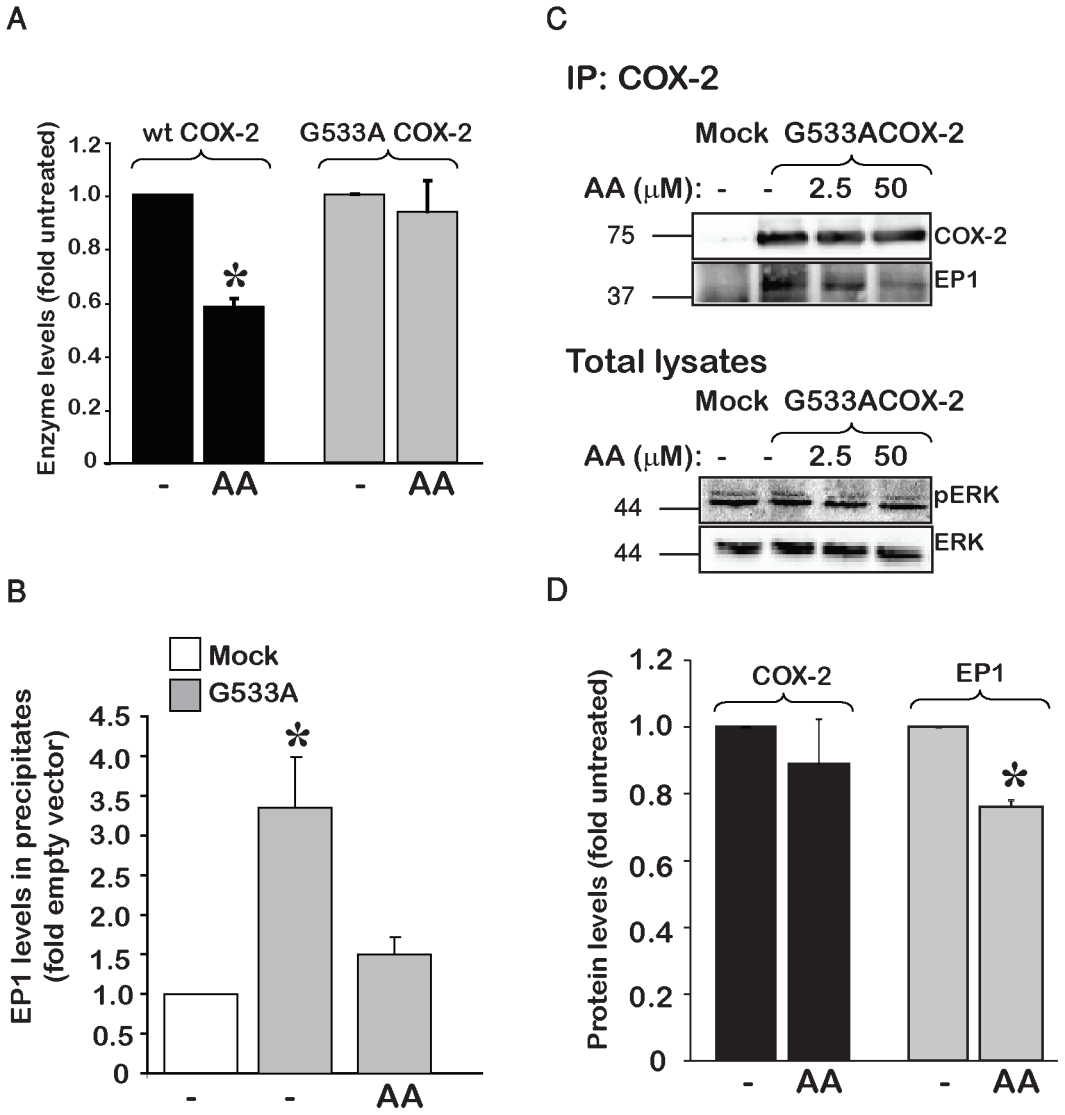
The interaction between COX-2 and EP_1_ depends upon the conformation of COX-2. *A*, Cells were transfected with either wild type (wt) or G533A COX-2 and exposed to 10 µM AA for 19 h. While wt COX-2 levels were lowered in the presence of AA, G533A COX-2 was not affected. n = 4 independent experiments *p<0.05 vs. untreated. *B*, Summary graph of n = 5 independent experiments showing a reduction in the levels of EP_1_ co-immunoprecipitated with COX-2 following treatment with AA. *p<0.05 vs. Mock transfection. HEK 293 cells, transfected with mock or G533A COX-2 were treated with either vehicle (ethanol) or AA (50 µM) for 15 min. Blots were probed for COX-2 and EP_1_. *C*, Representative immunoblot of n = 3 independent experiments depicting the dose-dependent effect of AA on the interaction of G533A COX-2 with EP_1_. Cells were transfected with either mock or G533ACOX-2 DNA, and 16 h post-transfection treated with 2.5 or 50 µM AA for 15 min prior to immunoprecipitation. Immunoprecipitate were probed for COX-2 and EP_1_ levels. Total lysates from the same samples were probed first for phosho-ERK followed by total ERK levels. *D*, prolonged exposure to AA, does not affect G533A COX-2 but reduces EP_1_ levels. Cells overexpressing G533A COX-2 were treated with 10 µM AA for 1.5–2 h, samples were collected and probed for levels of COX-2 and EP_1_. n = 3 independent experiments, *p<0.05 vs. untreated.

The use of wild type COX-2 to measure the interaction between the enzyme and the receptor does not discriminate whether the association between the two proteins is decreased because of direct binding of substrate, or whether it is an indirect effect of AA metabolites formed by the enzymatic activity of COX-2. To discriminate between the two possibilities, we employed a catalytically impaired COX-2 mutant, G533A COX-2 that was reported to bind AA but lack significant catalytic activity [18]. To ascertain that G533A is indeed devoid of catalytic activity we measured wild type and mutant COX-2 levels following overnight exposure to AA. In accordance with previous reports [13], prolonged exposure of wild type enzyme to AA caused a marked reduction in its levels. In contrast, the same incubation conditions had no effect the levels of G533A (Fig. 3A). We then performed the same co-immunoprecipitation experiments as in Fig. 2 using G533A COX-2. As in the wild type COX-2 experiments, EP_1_ was present in the G533A mutant precipitates (Fig. 3B). Similarly to the wild type enzyme, treatment with AA caused a significant reduction in the EP_1_ that was associated with the inactive enzyme (Fig. 3B), an effect which was apparent at 2.5 µM of AA and increased at 50 µM of AA stimulation (Fig. 3C). Measurements of phospho-ERK levels showed no activation of the MAP kinase pathway supporting the observation that G533A COX-2 is indeed incapable of producing a significant amount of prostaglandin products. Lastly, prolonged exposure of G533A COX-2 to AA did not cause a reduction in its levels but like the wild type enzyme caused a decrease in the expression of EP_1_ (Fig 3D). Together these data indicate that the decrease in association between EP_1_ and COX-2 is due to binding of AA to COX-2.

**Figure 4:**
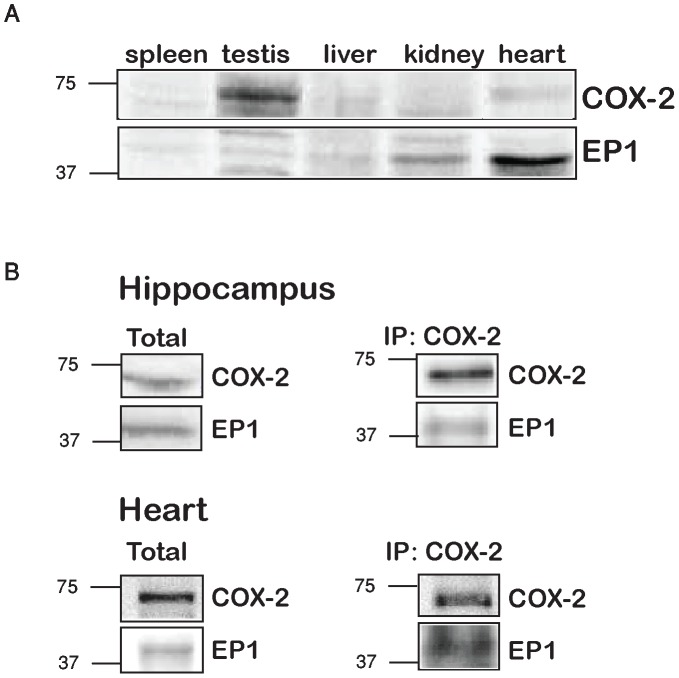
The interaction between COX-2 and EP_1_ is observed in rat tissues. *A*, Rat internal organs were harvested, homogenized and equal amounts of total protein lysates were separated by SDS-PAGE and probed for COX-2 and EP_1_ expression. *B*, COX-2 was immunoprecipitated from hippocampus (upper panel) and heart tissues (lower panel) and probed for COX-2 and EP_1_. Both tissues show the presence of EP_1_ in COX-2 precipitates.

Since the existence of a transient regulatory mechanism of expression control between COX-2 and EP_1_ may have a great physiological relevance, we tested whether the interaction between these two proteins occurs in mammalian tissue. Measurements of COX-2 and EP_1_ expression levels in lysates of rat internal organs (spleen, testis, liver, kidney and heart) revealed that they are both endogenously expressed in these tissues at different levels (Fig. 4A). To test for a possible interaction between COX-2 and EP_1_ *in vivo*, we precipitated COX-2 from rat hippocampus and heart and probed for the presence of EP_1_. As depicted in Fig 4B, EP_1_ was present in COX-2 precipitates of both hippocampal and heart tissues, suggesting that the interaction between EP_1_ and COX-2 also occurs in tissues.

## Discussion

The main observation of the present study is that elevation in COX-2 expression is accompanied by an increase in the levels of endogenous EP_1_ receptor via a mechanism that involves an interaction between the two proteins. Furthermore, disruption of the complex due to binding of AA to COX-2 is characterized by a reduction in EP_1_ expression. These findings are complementary to a phenomenon that we have reported recently, whereby an elevation in the levels of EP_1_ downregulates the expression of COX-2 in a pathway that does not involve activation of the receptor [15]. Since COX-2 ubiquitination is increased in the presence of EP_1_, the interaction between COX-2 and EP_1_ most likely involves additional proteins such as an E3 ligase and other scaffold proteins that are involved in accelerating COX-2 degradation. While the identity of the domains and proteins that are involved in the interaction is not yet known, our combined results suggest that a transient interaction between COX-2 and EP_1_ may be part of a feedback loop, whereby an increase in COX-2 expression (e.g. during inflammation) elevates EP_1_ by positive feedback, which ultimately acts to downregulate COX-2 by expediting its degradation. Such an interaction may serve as a post translational mechanism for controlling both the ligand generating system of COX-2 and its reception system, and act as a signaling modulator that responds to intra-and extracellular cues quickly and effectively.

Our data show that complex formation between the two proteins is important for the effect of COX-2 on EP_1_ because its disruption is accompanied by a reduction in EP_1_ levels. However, it does not provide a definite answer as to whether the interaction alone is sufficient to explain the increase in EP_1_ and not other EP receptors. In fact all four subtypes of EP receptors form complexes with COX-2 (data not shown), suggesting that the unique effect of COX-2 on EP_1_ requires additional, unknown factors that may stem from its structure and cellular localization. An examination of the structure of EP receptors reveals that while they share the property of higher affinity to PGE_2_ compared to other prostanoid receptors, they display very limited homology among themselves (∼30%), particularly in the intracellular third loop and C terminus that are involved in protein-protein interactions [4]. Compared to EP_2_, EP_3_ and EP_4_ that act on adenylate cycles via Gs and Gi proteins, the identity of the G protein that is activated by EP_1_ is not known, but seems to occur via Gq-coupled pathways [7], [8]. Furthermore, unlike most GPCRs, EP_1_ localizes in several intracellular structures including Golgi, and both membranes of the nuclear envelop [19], [20], [21], [22], where its stimulation causes a concentration-dependent rise in intra-nuclear calcium [19]. Together these finding suggest that the mechanism that underlies the upregulation of EP_1_ by COX-2 is complex and is only partly explained by an interaction between the two.

Contrary to the accepted paradigm that COX-2 is only overexpressed in tissues following pathological cues, we find that many rat organs express varying levels of COX-2 under normal conditions. These results are in accordance with other recent studies that demonstrated the presence of COX-2 in the parenchymal cells of many human and mouse tissues [23], [24]. We also show that in tissues such as the heart and the hippocampus, COX-2 is found in complex with EP_1_. While the physiological role of such a complex is still obscure, it is tempting to speculate that the interaction between the two proteins may participate in maintaining homoeostasis in these organs through reciprocal regulation. Therefore it is important to establish the relationship and dynamics of the complex in pathologies such as gastrointestinal, breast, prostate and lung malignancies, that are characterized by COX-2 overexpression [24], [25]. Furthermore, since the interaction between COX-2 and EP_1_ is transient and breaks down following binding of AA, it is possible that other non-substrate fatty acids (e.g. oleic, stearic), which allosterically modulate COX function [26], may have a regulatory role in modulating the EP_1_-COX-2 complex.

The role of COX-2 as a major mediator of the inflammatory response is unquestioned. While the signaling cascades that lead to the induction of COX-2 are well-characterized [13], there is much less information about pathways mediate its degradation. An interaction of COX-2 with caveolin-1 and EP_1_ reduces COX-2 expression by accelerating its ubiquitination and degradation [15], [27], [28]. The data presented herein suggests the existence of a feedback loop between COX-2 and EP_1_ that may provide an unappreciated means of controlling COX-2 and possibly constitute a novel therapeutic target in inflammatory disease. Identification of the specific protein domains that are involved in this interaction is a matter of current investigation.
